# Interactions between atrial fibrosis and inflammation in atrial fibrillation

**DOI:** 10.3389/fcvm.2025.1578148

**Published:** 2025-07-10

**Authors:** Zhihua Pang, Ying Ren, Zhuhua Yao

**Affiliations:** Department of Cardiology, Tianjin Union Medical Center, The First Affiliated Hospital of Nankai University, Tianjin, China

**Keywords:** atrial fibrillation, fibrillation, atrial, inflammation, fibrosis, atrial fibrosis

## Abstract

Atrial fibrillation (AF) is a complex arrhythmia driven by intricate pathophysiological mechanisms, with atrial fibrosis and inflammation emerging as central players in its initiation and perpetuation. Key pathways, including the renin-angiotensin-aldosterone system (RAAS), TGF-β/Smad signaling, and pro-inflammatory cytokine cascades (e.g., TNF-α/NF-κB, IL-6/STAT3), contribute to fibrotic remodeling and electrophysiological dysfunction. These pathways promote extracellular matrix deposition, fibroblast activation, and heterogeneous conduction, creating a substrate for AF maintenance. Contemporary therapeutic approaches predominantly target rhythm control via catheter ablation techniques and pharmacological interventions with antiarrhythmic agents. Nevertheless, the efficacy of anti-inflammatory approaches, such as corticosteroids and colchicine, remains uncertain due to limited robust clinical evidence, highlighting the need for further investigation. Advanced fibrosis quantification modalities, particularly late gadolinium-enhanced magnetic resonance imaging and electroanatomic mapping, have emerged as valuable tools for optimizing ablation strategies. Furthermore, emerging evidence highlights significant sex-based disparities in atrial fibrosis distribution and electrophysiological substrate characteristics, suggesting the potential for gender-specific therapeutic approaches. This comprehensive review systematically examines the pathophysiological roles of atrial fibrosis and inflammation in AF progression, with particular emphasis on their intricate bidirectional relationship. Through detailed elucidation of these mechanistic interactions, we aim to facilitate the development of novel therapeutic interventions to enhance clinical management of AF.

## 1 Introduction

Atrial fibrillation (AF) is the most common arrhythmia in clinical practice, characterized by irregular atrial electrical activity and ineffective atrial contractions, leading to decreased cardiac function. More than 37.5 million people worldwide suffer from AF. In the past 20 years, the global incidence and prevalence of AF have both increased by more than 30%, and it is expected to continue to increase in the next 30 years (1). In China, there are approximately 7.9 million patients with AF, with a weighted prevalence of 1.8% (2). AF can significantly increase the risk of death, stroke, heart failure (HF), cognitive dysfunction, and dementia, seriously affecting the quality of life of patients (3–5), and causing a huge burden on the health and economy of patients. Catheter ablation and antiarrhythmic drugs (e.g., amiodarone and flecainide), have emerged as cornerstone therapeutic modalities for atrial fibrillation (AF) management (6).

As a complex heterogeneous arrhythmia, the occurrence, persistence, and occurrence of complications of AF involve multiple factors. The main pathogenesis of AF includes the presence of atrial ectopic electrical activity and reentry, involving atrial electrophysiological and structural remodeling (7, 8). Atrial fibrosis is a characteristic change in atrial structural remodeling, which can cause heterogeneous conduction in the atrium, leading to unidirectional conduction block and reentry, thereby triggering AF. At the same time, long-term AF can exacerbate atrial fibrosis, further promoting the progression and maintenance of AF, known as “AF promoting AF” (9, 10). Inflammation is involved in the pathological process of various cardiovascular diseases and is the main regulatory factor of repair response after cardiac injury (11, 12). A large amount of evidence supports the close relationship between inflammation and AF. The atrial electrophysiological and structural remodeling mediated by inflammatory response are important risk factors for inducing AF, and the activity of AF itself can also induce inflammatory response, forming the “AF promoting AF” cycle (13, 14). Since both fibrosis and inflammation play important roles in atrial remodeling, what is the relationship between the two? The crosstalk between fibroblasts and immunocytes demonstrates the interaction between fibrosis and inflammation, which together promote atrial remodeling, leading to the occurrence and persistence of AF (15). This article will review the roles of atrial fibrosis and inflammation in the pathophysiological mechanisms of AF, their relationship, and corresponding treatment methods to provide a theoretical basis for the clinical management of AF.

## 2 Atrial fibrosis and AF

### 2.1 Atrial fibrosis

Atrial remodeling plays a central role in the occurrence and development of AF, and atrial fibrosis is one of the key factors in atrial remodeling (16). Atrial fibrosis is a process of cardiac remodeling caused by the interaction of multiple neurohormonal mediators, characterized by abnormal activation, proliferation, and differentiation of cardiac fibroblasts, as well as excessive deposition of extracellular matrix (ECM) proteins (17). Fibroblasts are the main cells that regulate the synthesis and composition of ECM. Fibroblasts are the most numerous cells in the heart, accounting for approximately 75% of all heart cells (18). When various harmful stimuli cause myocardial injury, fibroblasts migrate to the damaged area, proliferate, and transform into the phenotype of myofibroblasts. The contractility of myofibroblasts is enhanced through the secretion of contractile proteins such as alpha-smooth muscle actin (*α*-SMA), which participate in cardiac injury repair. However, sustained damage may overactivate fibroblasts, causing them to continuously synthesize ECM, leading to excessive deposition of ECM, collagen proportional imbalance, especially the increase in the proportion of type I and III collagen, and disordered collagen alignment, ultimately developing into progressive fibrosis (19–21).

Myocardial fibrosis is divided into two different types, namely reparative fibrosis and interstitial fibrosis. Reparative fibrosis refers to the replacement of necrotic cardiomyocytes with fibrosis tissue, with the most obvious example being myocardial infarction (MI) scars. Interstitial fibrosis refers to the abnormal accumulation of ECM around the interstitium and blood vessels without significant cardiomyocyte loss, which is more common in non-ischemic cardiomyopathy (22–24).

Atrial fibrosis is typically considered a type of myocardial fibrosis. But in fact, this view is problematic. The experimental results on congestive heart failure (CHF) canine model indicated that the AF substrate of CHF was associated with widespread cell death (25) and fibrosis disruption of muscle bundle continuity (26), leading to longitudinal conduction disorders. Another study has shown that the thicker the left atrial interstitial collagen strands in patients with AF, the longer the duration of AF, and the faster the longitudinal conduction velocity. This suggested that the structure and severity of AF were related to atrial conduction abnormalities (27). Therefore, for atrial fibrosis that occurs in AF, these two different types of fibrosis may coexist.

### 2.2 Relationship between atrial fibrosis and AF

AF is a complex and progressive disease that requires triggering and susceptible substrates for its occurrence and maintenance. The current research has found that the triggering sites of AF mainly include the atrial sleeves of the pulmonary veins (PVs) and long-standing rotors with fibrillatory conduction. Intracellular Ca^2+^ handling and autonomic nerve activation can promote early afterdepolarization (EAD) and delayed afterdepolarization (DAD) activities, which induce ectopic focal discharges in PVs, leading to AF (28, 29). And the rotor is another possible trigger site for AF, which is composed of heterogeneity in the form of spatially distributed refractory gradients in the atrium. The waves emitted by the high-speed rotation of the rotor can cause turbulent electrical activation, manifested as fibrillatory conduction, thereby triggering AF (28, 30).

In addition, atrial fibrosis, as a susceptible substrate to AF, plays a crucial role in the sustained development of AF. The landmark DECAAF study, a multicenter prospective observational cohort investigation involving 260 patients with both paroxysmal and persistent AF, demonstrated a significant correlation between atrial fibrosis extent and AF recurrence risk. The quantitative assessment of atrial fibrosis in patients showed that for every 1% increase in fibrosis degree, the risk of AF recurrence increased by 6%. The degree of atrial fibrosis was an independent predictor of AF recurrence (31). Additionally, extensive preclinical studies using various animal models have further substantiated the pivotal role of atrial fibrosis in AF initiation and maintenance. Both HF and chronic mitral regurgitation (MR) dog models exhibited significant interstitial fibrosis, which induced and maintained AF by causing local conduction interference (32, 33). In a goat model with cardiac specific overexpression of transforming growth factor beta 1 (TGF-β1), increased atrial fibrosis, progressive P-wave prolongation, and slowed atrial conduction were observed, leading to increased AF susceptibility (34). Meanwhile, a study on a transgenic mouse model of atrial fibrosis induced by TGF-β1 overexpression demonstrated that fibrosis could enhance atrial conduction heterogeneity, making reentry more likely to occur, thereby promoting the progression and maintenance of AF (35). The above studies involving patients, large animal models, and transgenic animal models showed that atrial fibrosis increased AF susceptibility and the risk of AF recurrence. Atrial fibrosis can cause and maintain AF by altering the atrial conductibility, leading to local conduction block and reentry.

In fact, atrial fibrosis may also be a result of AF. Clinical pathological examinations reveal that approximately 17% of patients with lone AF demonstrate patchy fibrosis patterns on atrial biopsy (36). Additionally, experimental investigations utilizing canine rapid atrial pacing models revealed significantly augmented interstitial fibrosis in AF-induced animals relative to control cohorts (37). Similarly, a dog model study aimed at exploring the impact of AF on electrophysiology showed that AF without ventricular dysfunction lead to atrial fibrosis and increased susceptibility to AF, while AF with rapid ventricular response increased atrial and ventricular fibrosis (38). In summary, atrial fibrosis is both a triggering factor and a result of AF, playing a crucial role in its occurrence and sustained development.

### 2.3 Profibrotic substrate

**Figure 1:**
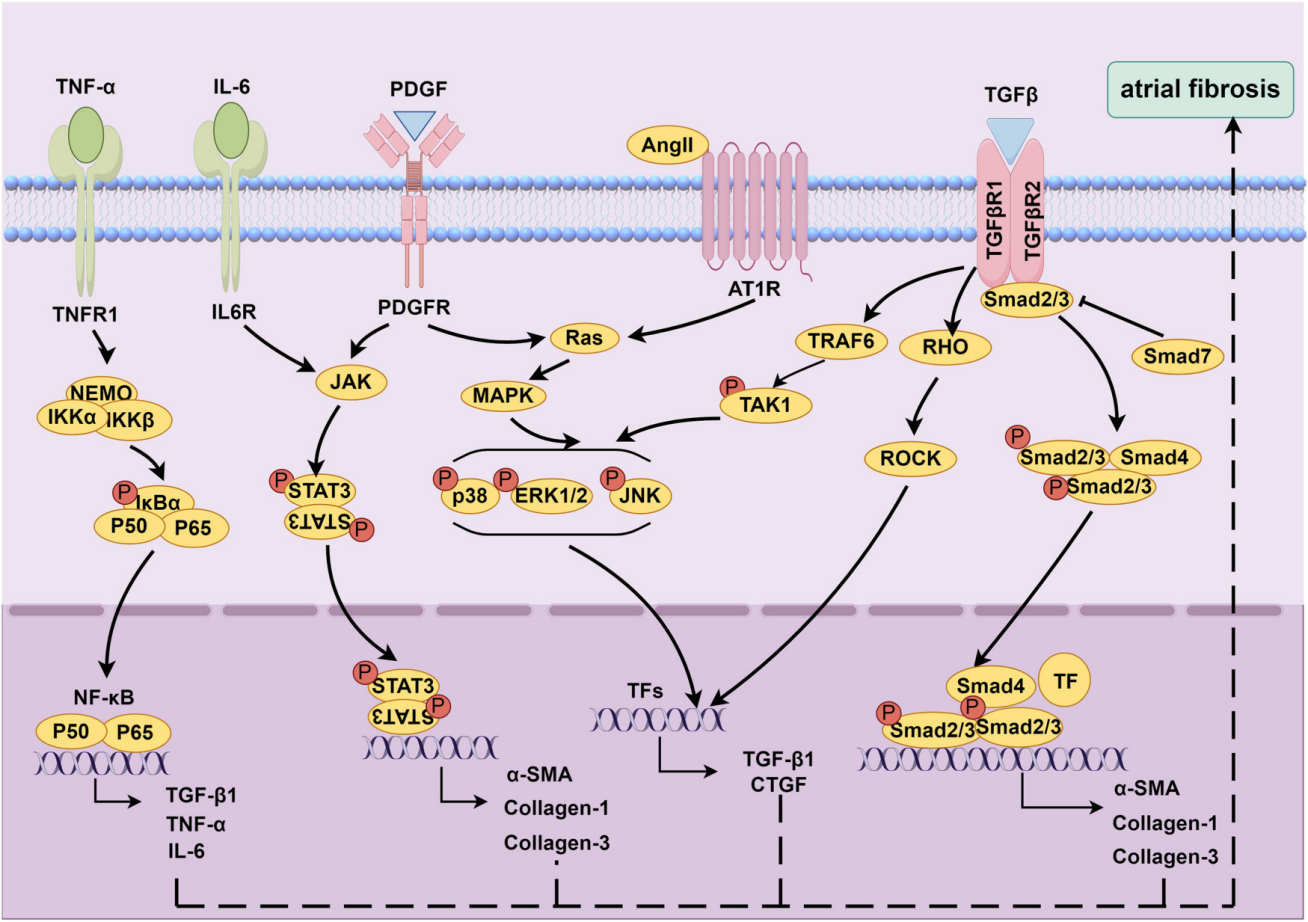
Molecular mechanisms underlying atrial fibrosis. Created using Figdraw, www.figdraw.com.

Major contributors to advancing atrial fibrosis and their mechanistic pathways are summarized (Figure 1).

#### 2.3.1 Renin-angiotensin-aldosterone system (RAAS)

RAAS is a hormone cascade reaction primarily responsible for regulating blood pressure and water-salt balance, maintaining homeostasis in the human body (39). RAAS is involved in the fibrosis process of various diseases, such as hypertension (40), CHF (41), and MI (42). Angiotensin II (Ang II) is a key molecule in this system and plays an important role in atrial fibrosis (39, 43, 44). A study suggested that the occurrence of atrial fibrosis in CHF dog models was associated with increased Ang II concentration (44). Moreover, a mouse model overexpressing angiotensin converting enzyme (ACE) showed atrial dilation, focal fibrosis, and AF (43). In addition, blocking the effect of Ang II with ACE inhibitors (ACEIs) can reduce atrial fibrosis (44, 45). Regarding the mechanism of Ang II promoting fibrosis, previous studies have confirmed that after binding to Angiotensin II type 1 receptor (AT1R), Ang II stimulated fibroblast proliferation and differentiation by activating the phosphorylation cascade of mitogen-activated protein kinase (MAPK) (46, 47). After activating the MARK cascade with Ang II, atrial fibrosis could be mediated by upregulating the expressions of TGF-β1 (48–52) and connective tissue growth factor (CTGF) (49, 53, 54).

#### 2.3.2 TGF-β1

TGF-β1 is an important profibrotic cytokine. TGF-β1 can mediate the differentiation of fibroblasts into myofibroblasts and promote increased collagen secretion by activating Smad dependent or independent signaling pathways (7, 9, 55). In the classic Smad dependent signaling pathway, TGF-β1 binds to type I and type II receptors, activating downstream Smad2/3/4 proteins and promoting increased collagen secretion (56). TGF-β1 can also reduce the negative feedback regulation of TGF -β1/Smad signaling by inhibiting Smad7 (57, 58). The currently discovered Smad independent signaling pathways mainly include the MAPK/TGF-β1/tumor necrosis factor receptor associated factor 6 (TRAF6)/TGF-β activated kinase 1 (TAK1) signaling (59, 60) and TGF-β1/Ras homolog family member A (RhoA)/Rho-associated kinase (ROCK) (61).

#### 2.3.3 Cytokines

Inflammatory response is closely linked to the formation of atrial fibrosis (7, 9, 55). Multiple inflammatory cytokines, such as tumor necrosis factor alpha (TNF-α), interleukin (IL) -1β, IL-2, IL-6, etc., can mediate the occurrence of atrial fibrosis. Liew et al. found that TNF-α activated fibroblasts and promoted collagen synthesis by activating the TGF-β signaling pathway and promoting the secretion of matrix metalloproteinases (MMP), thereby mediating the occurrence of atrial fibrosis in mice (62). Inhibition of the TNF-α/nuclear factor-kappaB (NF-κB)/TGF-β signaling pathway can effectively suppress myocardial fibrosis and cardiac remodeling, thereby attenuating the progression of AF (63). Meanwhile, the activation of signal transducer and activator of transcription 3 (STAT3) signaling pathway by IL-6 contributes to AF development through stimulating cardiac fibroblast activation (64). Chen et al. found that IL-6-miR-210 promoted the expressions of α-SMA, type I collagen, and type III collagen by targeting Foxp3, leading to atrial fibrosis (65). Studies have shown that epicardial adipose tissue (EAT) could secrete pro-inflammatory cytokines such as TNF-α, IL-1, IL-6, and monocyte chemoattractant protein-1 (MCP-1), which could trigger inflammation in adjacent atrial tissue through paracrine action, leading to atrial fibrosis (66–69).

#### 2.3.4 PDGF

The platelet-derived growth factor (PDGF) family proteins are encoded by four genes, namely PDGF-A, PDGF-B, PDGF-C, and PDGF-D (70). PDGF can promote proliferation and differentiation of fibroblasts, and regulate ECM synthesis via various pathways, such as MAPK, Janus kinase (JAK)/STAT, and Ras/extracellular regulated protein kinase 1/2 (ERK1/2) (55). Different subtypes of PDGF are involved in the development of myocardial fibrosis. Studies have shown that PDGF-D promoted the proliferation and differentiation of rat cardiac fibroblasts, as well as the secretion of type I collagen, by mediating the activation of TGF-β1 signaling pathway, exerting a profibrotic effect (71). Cardiac fibrosis was observed in mice with cardiac specific overexpression of PDGF-A and PDGF-B (72). Liao et al. found that the expression of PDGF-A increased in mast cells in the atrium of mice with pressure-overloaded heart, promoting fibroblast proliferation and collagen synthesis, thereby promoting atrial fibrosis and enhancing susceptibility to AF (73). In the HF dogs induced by rapid ventricular pacing, the mRNA levels of PDGF subtypes A, C, and D in the left atrial (LA) fibroblasts increased, activating the JAK-STAT pathway, promoting ECM synthesis and LA fibrosis (74). In addition, in rat cardiac allografts, these PDGF subtypes mediated profibrotic effects by regulating the TGF-β1 signaling (75).

#### 2.3.5 miRNA

Micro-ribonucleic acids (microRNAs, miRNAs or miRs) are a class of evolutionarily conserved non-coding small molecule RNAs, typically between 21 and 23 nucleotides in length, that can regulate gene expression at the translation level (76). Multiple studies have shown that miRNA plays an important role in atrial fibrosis and AF (22, 55, 77). Among them, miR-21 is a promising target that regulates AF and atrial fibrosis through multiple mechanisms. In a rat model of HF induced AF, the expression of atrial miR-21 was upregulated, and knocking it out could inhibit atrial fibrosis and AF development (78). The research of Adam et al. showed that compared with sinus rhythm (SR) population, miR-21 expression was upregulated in LA of AF patients. After Rac1 was activated by Ang II, the expressions of CTGF and lysyl oxidase increased, mediating the increase in miR-21 expression and the decrease in its downstream molecule Sprouty 1 (Spry 1, a protein that inhibits fibroblast proliferation) expression, leading to an increase in atrial collagen content and promoting fibrosis (79). Another study has shown that miR-21 was upregulated in fibroblasts of failing hearts and activated the ERK-MAPK signaling pathway by inhibiting Spry1, thereby promoting fibroblast proliferation and interstitial fibrosis (80). He et al. found that in a rabbit model of AF induced by rapid atrial pacing, miR-21 could also reduce the inhibitory feedback regulation of TGF-β1/Smad signaling by mediating Smad7 specific degradation, thereby promoting the development of atrial fibrosis in AF (81). In addition, in a rat model of sterile pericarditis, STAT3 and miR-21 formed a feedback loop, promoting fibroblast proliferation and increasing ECM synthesis, thereby increasing AF susceptibility (64). Other miRNAs are also involved in the process of atrial fibrosis.

For example, elevated miR-486-5p levels were detected in AF patients and correlated with increased left atrial fibrosis occurrence (82). The downregulation of miR-26 regulated the inward-rectifier potassium current in fibroblasts by increasing KCNJ2 expression, thereby promoting fibroblast proliferation and AF (83, 84). Wang et al.'s study showed that downregulation of miR-27b inhibited the Smad2/3 signaling by targeting ALK5, thereby improving Ang II induced atrial fibrosis and AF (85). MiR-29b may be involved in atrial fibrosis. In a canine model of CHF induced by rapid ventricular pacing, miR-29b expression was reduced in atrial tissue and atrial fibroblasts, accompanied by increased ECM expression in fibroblasts (86). MiR-30 and miR-133 could reduce collagen production and inhibit cardiac fibrosis by downregulating CTGF (87).

## 3 Inflammation and AF

### 3.1 Relationship between inflammation and AF

Numerous studies have shown that inflammation is involved in the occurrence and development of various cardiovascular diseases (88, 89). Regarding the link between inflammation and AF, Bruins et al. first discovered that C-reactive protein (CRP) level in patients with coronary artery disease (CAD) was associated with arrhythmia after revascularization (90). Afterwards, Chung et al. also found that serum CRP levels in patients with AF were higher than those in patients with SR, and CRP levels in patients with persistent AF were higher than those in patients with paroxysmal AF (91). Both studies suggest that inflammatory response is closely related to AF. With the continuous exploration of the relationship between inflammation and AF, the causal relationship between the two is gradually becoming clear.

#### 3.1.1 Pathological mechanisms of inflammation promoting AF

Inflammatory response triggers and maintains AF by altering the electrophysiology and structure of atrial tissue, leading to atrial electrical and structural remodeling (13, 14, 92).

##### 3.1.1.1 Electrical remodeling mechanisms

Regarding atrial electrical remodeling, multiple studies have shown that various inflammatory factors, such as TNF (93–96) and PDGF (97), as well as NLRP3 inflammasome (98–100), can induce atrial electrical remodeling by inducing abnormal calcium processing, triggering abnormal PV electrical activity, shortening the atrial action potential duration, leading to inflammation related AF. Moreover, the abnormal expression and distribution of atrial connexin 40 (Cx40) and Cx43 caused by inflammatory response can induce atrial heterogeneous conduction, which is an important factor in increasing susceptibility to AF (101). Studies have shown that TNF-α (102) and IL-6 (103) can cause downregulation of Cx40 and Cx43 expression, leading to abnormal atrial conduction and inducing atrial electrical remodeling. In addition, NF-κB, a transcription factor that regulates the expression of multiple inflammatory cytokines, can induce downregulation of Na^+^ channel expression by binding to Na^+^ channel promoter region, leading to atrial electrical remodeling in AF (104).

##### 3.1.1.2 Structural remodeling mechanisms

In terms of atrial structural remodeling, various inflammation associated cytokines, such as TNF-α (62), IL-6 (65), PDGF (73, 105), galectin-3 (106), etc., can also induce the occurrence and development of AF by promoting atrial fibrosis. TNF-α induces atrial fibrosis and alters Cx40 expression by regulating the TGF-β/Smad signaling, activating fibroblasts, and promoting MMP secretion, thereby promoting the development of AF in mice (62). IL-6 can also activate the TGF-β/Smad signaling pathway, leading to cardiac fibrosis (107). In addition, a large number of immunocytes in atrial tissue can also mediate the profibrotic process (7, 108). After cardiac injury, macrophages can induce the migration, proliferation, and activation of fibroblasts, and promote ECM synthesis by producing various pro-inflammatory cytokines (such as TNF-α and IL-6), profibrotic cytokines (such as TGF-β and PDGF), and profibrotic proteases (such as MMP and chymase), thereby exerting profibrotic effects (109–111). Similarly, studies have shown that neutrophils, T cells, and mast cells also participate in the profibrotic process (108, 110).

#### 3.1.2 Feedback mechanisms by which AF exacerbates inflammation

Conversely, AF can also induce inflammation, thereby further promoting the development of AF (13, 14, 112). A prospective study on patients with persistent AF found that after restoring and maintaining SR, the levels of high-sensitivity CRP (hs-CRP) in AF patients were significantly reduced [0.10 (SD 0.06) mg/dl vs. 0.29 (SD 0.13) mg/d1, *p* < 0.001] (113). Another case-control study of AF patients also showed that the levels of CRP (3.1 mg/dl vs. 1.7 mg/dl) and IL-6 (2.3 ng/ml vs. 1.5 ng/ml) were higher during AF than during SR (114). In addition, a prospective study on patients with atrial flutter also found that after radical ablation, the levels of CRP (6.28 mg/L vs. 2.92 mg/L, *p* = 0.028) and IL-6 (*p* = 0.002) in patients with atrial flutter significantly decreased (115). The above clinical studies all indicate that AF is the cause of inflammation, not the result.

In a rapid atrial pacing induced AF dog model, we observed elevated levels of TNF-a, IL-6, and CRP, shortened effective refractory period, and increased AF susceptibility (116, 117). The anti-inflammatory effect of prednisone could effectively reverse this process and significantly shorten the AF duration (118). However, the specific mechanism by which AF leads to inflammation is currently unclear. Some studies suggested that AF may trigger calcium overload in atrial myocytes, leading to programmed cell death and the release of danger-associated molecular patterns (DAMPs) to activate low-grade inflammatory responses to repair cell damage (112, 119). A study evaluating the relationship between cell free DNA (cfDNA) and AF found that in the AF HL-1 cell model, unmethylated mitochondrial cfDNA (mt-cfDNA) promoted the expression of IL-1β and IL-6, indicating that AF could induce systemic inflammation through cfDNA (120). Further in-depth research is needed on the molecular mechanisms underlying AF induced inflammation.

From this, it can be concluded that inflammation may lead to AF, and AF can also promote inflammation, forming a vicious cycle.

### 3.2 Systemic inflammation and AF

Many systemic diseases are associated with low-grade inflammation, which may be the source of AF associated inflammation (5, 7, 112).

#### 3.2.1 Severe sepsis

The incidence rate of AF in sepsis patients is high (121–123). Meierhrich et al. found that CRP levels in septic shock patients remained consistently and significantly elevated before the onset of AF, which can prove that systemic inflammation is an important factor in triggering AF (122).

#### 3.2.2 Chronic inflammatory diseases

The risk of AF was significantly increased in patients with rheumatoid arthritis (RA) (124–126). Although the underlying mechanism of RA induced AF is complex, the key factor is still systemic inflammatory response. Systemic inflammation activation can not only produce substrates for promoting AF by accelerating the development of ischemic heart disease (IHD) and CHF, but also directly trigger AF by altering atrial electrophysiology (127). A clinical study involving over 20,000 patients with autoimmune rheumatic disease (ARD) showed that high CRP level was an independent predictor of AF in ARD patients (HR 1.75, 95%CI 1.07–2.86, *p* = 0.04), indicating that the risk of AF in ARD patients was influenced by inflammatory responses (128). In addition, it was found in a rat model of RA that the inducibility and duration of AF were obviously increased, and the AF duration was significantly positively correlated with serum IL-6 and TNF-α levels, indicating that RA related systemic inflammation was associated with increased susceptibility to AF (129). Psoriatic patients, especially those with psoriatic arthritis, have an increased risk of developing AF (130, 131). The risk of AF was also significantly increased in patients with inflammatory bowel disease (IBD) (132, 133). A study has found that the P-wave dispersion in IBD patients, a risk factor for the development of AF, was significantly higher than that in healthy individuals (134). Another study has shown that atrial electrical conduction was delayed in IBD patients, and chronic inflammation activation might induce electrophysiological and structural changes in atrial tissue, which is the main factor leading to slowed atrial conduction velocity (135).

#### 3.2.3 Hypertension

Hypertension is an independent risk factor for AF (136–138). Ang II is a key molecule in the RAAS system and a major mediator of hypertensive vasoconstriction. It can trigger systemic inflammatory response by stimulating the production of inflammatory cytokines, activating immunocytes, and promoting immunocyte recruitment (139). Hypertension related inflammation can induce atrial electrical and structural remodeling, thereby triggering and maintaining AF. In hypertensive sheep and rat models, an increase in atrial inflammatory infiltration was observed, which was associated with the occurrence of atrial fibrosis and remodeling (140, 141). The pathogenesis of hypertension related inflammation induced AF needs further clarification.

#### 3.2.4 Metabolic disorders

Obesity is an important risk factor for new-onset AF in the general population and patients after cardiac surgery (142–144). Obesity can not only induce immunocyte activation and infiltration into adipose tissue (145–147), but also promote the secretion of a large number of inflammatory cytokines (148, 149). The resulting low-grade systemic inflammatory response may lead to the occurrence and development of AF (150, 151). Diabetes is also an important risk factor for AF (152, 153). Inflammation in the context of diabetes can participate in atrial electrical and structural remodeling, thus inducing AF (152, 154).

#### 3.2.5 CAD

CAD is an important risk factor for AF (155). Some studies suggested that chronic low-grade inflammatory response caused by CAD may be a triggering factor for AF. Stellos et al. found that there were differences in the expressions of platelet-bound stromal cell-derived factor-1 (SDF-1) and plasma SDF-1 between AF patients and SR population in CAD patients, and SDF-1 was associated with inflammatory cell recruitment (156). A clinical study found that IL-6 upregulation was significantly associated with the occurrence of AF in CAD patients, indicating that IL-6 is an important biomarker for CAD associated AF (157).

#### 3.2.6 Cardiac surgery and ablation

The systemic inflammatory response after cardiac surgery and radiofrequency catheter ablation is associated with the occurrence and recurrence of AF (158). The ARMYDA-3 study showed that postoperative high CRP level in patients receiving cardiac surgery was associated with an increased risk of AF (159). Another clinical study showed that IL-2 level in patients undergoing coronary artery bypass grafting (CABG) was associated with early postoperative AF (160). In a dog model of cardiac surgery induced inflammation, it was observed that the degree of atrial inflammation was associated with the inhomogeneity of atrial conduction and increased AF duration, which may be a factor in the early postoperative AF (161). In addition, multiple studies on the recurrence of AF after catheter ablation have confirmed that inflammatory biomarkers can serve as predictive factors for early recurrence of AF (162–164).

### 3.3 Inflammatory markers and AF

Inflammatory markers can predict the risk of AF and the prognosis of AF after cardioversion or ablation (165–167).

#### 3.3.1 CRP

CRP is an acute inflammatory protein commonly used as a biomarker for infection and inflammation in clinical practice (168, 169). CRP, including hs-CRP, is currently one of the most extensively studied inflammatory biomarkers for AF. Chung et al. found that compared to individuals with SR, CRP levels were elevated in patients with AF, and CRP levels were higher in patients with persistent AF than in patients with paroxysmal AF (91). Another study by Chung et al. showed that CRP is not only associated with the presence of AF, but can also predict the risk of AF in the future (170). Studies have shown that elevated CRP was significantly associated with an increased risk of mortality in patients with AF (171). CRP can also predict the risk of recurrence of AF after electrical cardioversion, catheter ablation, or cardiac surgery (166, 167, 172–174). In addition, hs-CRP is also associated with the occurrence and persistence of AF. Studies have shown that hs-CRP level is an independent predictor of successful AF cardioversion and SR maintenance after cardioversion (175, 176).

#### 3.3.2 Interleukins

Interleukin is a type of cytokine secreted by lymphocytes, macrophages, and other cells, which plays an important role in inflammatory responses (177). Among them, IL-6 has been relatively extensively studied in the field of AF. It was found that the increase of IL-6 was related to the increase of incidence of AF (157). Elevated IL-6 was significantly associated with increased risk of mortality in patients with AF (171). In addition, an increase in IL-6 was also associated with the prothrombotic state of AF (178). There is evidence to suggest that IL-6 could be used to predict the risk of AF after CABG (179) and the risk of AF recurrence after catheter ablation (166). Amdur et al. also found that plasma IL-6 level was an independent predictor of AF in patients with chronic kidney disease (CKD) (180). Other interleukins have also been shown to be associated with the occurrence and development of AF. Hak et al. found a direct correlation between serum IL-2 levels and AF after CABG, and IL-2 could serve as a predictive indicator for early AF after CABG (160). Moreover, serum IL-2 level could be used to predict the risk of AF recurrence after cardioversion or ablation (181, 182). Li et al. found that the level of IL-8 in the serum of patients with AF was elevated (183). Liuba et al. found that plasma IL-8 levels in the femoral vein, right atrium, and coronary sinus were elevated in patients with permanent AF compared to those with paroxysmal AF (184). Studies have shown that IL-8 was a predictive factor for new-onset AF in CAD patients after CABG (185–187). In addition, there is evidence to suggest that IL-1, IL-10, IL-18, etc. are also associated with AF (92, 112).

#### 3.3.3 TNF-α

TNF-α is a multifunctional pro-inflammatory cytokine that plays an important role in local and systemic inflammatory responses (188). Compared with individuals with SR, patients with AF had elevated levels of TNF-α (189), and the same phenomenon has also been observed in the context of valvular disease (190). In addition, the levels of TNF-α increased sequentially in patients with paroxysmal, persistent, and permanent AF (183). The above studies all indicate a close correlation between TNF-α levels and AF.

#### 3.3.4 Immunocyte population

White blood cell (WBC) count and neutrophil-to-lymphocyte ratio (NLR) are also common biomarkers of AF inflammation. Weymann et al. found that both WBC count and NLR were potential predictors for new-onset and recurrent AF (191). The Framingham Heart Study results showed a significant correlation between an increase in WBC count and AF events (192). Studies have shown that an increase in WBC count was an independent predictive factor of AF after cardiac surgery (193–195). In addition, after electrical cardioversion for persistent AF, the WBC count of patients maintaining SR was significantly reduced compared to those with early AF recurrence (196). And NLR can not only predict the risk of new-onset AF, but also predict the risk of recurrence of AF after cardiac surgery, radiofrequency ablation, and cardioversion (197–199).

#### 3.3.5 Others

MCP-1 is also an important pro-inflammatory cytokine that plays a crucial role in the occurrence and development of inflammation (200). Studies have shown that MCP-1 level was significantly increased in patients with AF (183, 201). Myeloproxidase (MPO) is a heme-containing protease secreted by neutrophils, which can participate in regulating the body's inflammatory response (202). There were studies confirming that patients with high MPO levels in paroxysmal AF had an increased risk of AF recurrence after catheter ablation (164, 203). Heat shock protein (HSP) is an important molecular chaperone protein in the body that can exert anti-inflammatory effects to protect the body from inflammatory damage (204). Currently, research has found that HSP27 and HSP70 can predict postoperative AF recurrence, and their mechanisms may be related to inflammation (205–207).

## 4 Relationship between atrial fibrosis and inflammation

**Figure 2:**
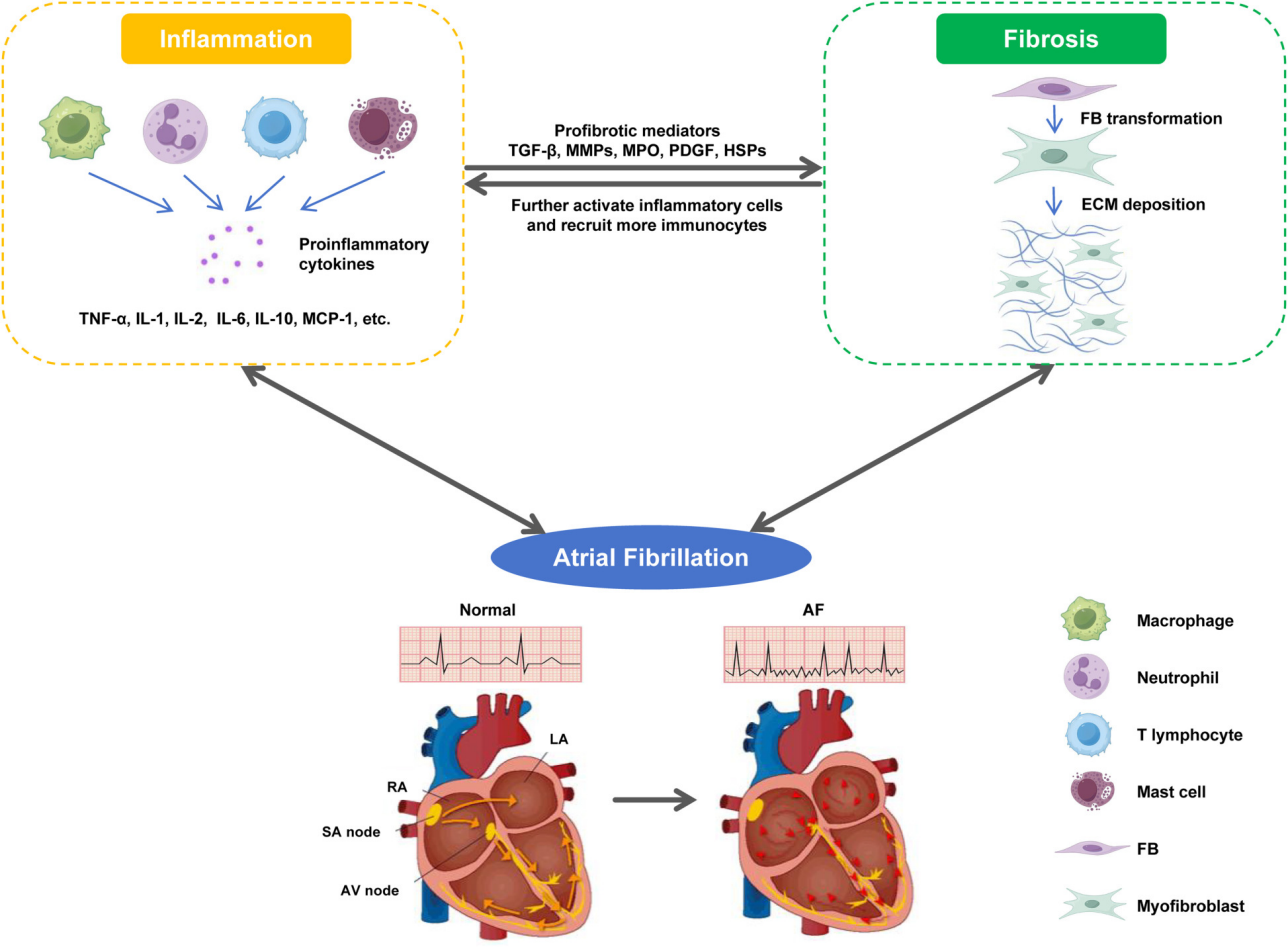
The malignant cycle between fibrosis and inflammation. In damaged heart, inflammatory cells trigger the phenotypic transformation of FBs into myofibroblasts by releasing a large amount of pro-inflammatory cytokines; Conversely, myofibroblasts produce a large amount of collagen and chemokines, which further activate inflammatory cells, and recruit and activate more immunocytes to enhance the cardiac inflammatory response. The malignant cycle between fibrosis and inflammation jointly triggers and maintains AF.

As the two main factors that induce and maintain AF, atrial fibrosis and inflammation are closely related (208, 209). As mentioned earlier, various pro-inflammatory cytokines and activated immunocytes can mediate the occurrence of atrial fibrosis through multiple mechanisms (7, 62, 108). Activated cardiac fibroblasts during fibrosis can also enhance local inflammatory responses by releasing inflammation associated cytokines and growth factors, and recruiting and activating more immunocytes (210, 211). At the level of molecular mechanism, the crosstalk between fibroblasts and immunocytes provides a good explanation for the self-sustaining relationship between fibrosis and inflammation: in damaged hearts, inflammatory cells can trigger the proliferation and differentiation of fibroblasts into myofibroblasts by releasing a large amount of inflammatory mediators; Conversely, myofibroblasts can also produce a large amount of collagen and chemokines, which further activate inflammatory cells and attract other immunocytes to enhance cardiac inflammatory response (15, 212, 213). In addition, there is clinical evidence supporting the view that there is a link between atrial fibrosis and inflammation. A study on evaluating left atrial remodeling in non-valvular AF showed that compared with SR patients, AF patients had significantly higher levels of NLR and hs-CRP, and NLR showed a highly significant correlation with LA volume index, indicating that AF inflammatory markers were associated with atrial remodeling (214). From this, it can be seen that inflammation leads to atrial fibrosis, and atrial fibrosis enhances local inflammatory response, forming a vicious cycle that synergistically increases the risk of AF (Figure 2).

Current studies have demonstrated that both atrial fibrosis and inflammatory responses are significantly associated with the risk of AF recurrence after ablation therapy (215, 216). From a pathophysiological perspective, the vicious cycle formed between inflammatory mediators and fibrotic progression may serve as a critical underlying factor contributing to poor clinical outcomes. A deeper understanding of this interaction mechanism may hold important clinical significance for the future development of multi-target combination therapeutic strategies (such as combined anti-inflammatory and anti-fibrotic therapies), potentially offering novel treatment approaches to improve long-term prognosis in patients with AF.

## 5 Detection tool for atrial fibrosis

We have previously pointed out that atrial fibrosis is the core pathophysiologic basis for the occurrence and maintenance of AF. Mechanistically, atrial fibrosis constitutes a potential substrate for arrhythmogenesis in AF, which leads to slowing and blocking of electrical conduction, increasing conduction heterogeneity, and formation of reentrant circuits, creating conditions for arrhythmia (217, 218). The triggered activities and arrhythmogenic substrates may interact to jointly promote the occurrence and persistence of AF (9, 55). Clinical evidence also demonstrated that the presence and severity of atrial fibrosis are closely related to poor clinical outcomes in AF patients. A retrospective study found that more severe LA fibrosis significantly increased the risk of stroke and transient ischemic attack in AF patients (219). Moreover, for AF patients undergoing catheter ablation, the degree of LA fibrosis was positively correlated with increased risk of recurrent arrhythmia and increased demand for repeat ablation (31, 220–222). Therefore, how to accurately detect and quantify atrial fibrosis has become an important issue in clinical diagnosis and treatment of AF. Currently, the main means used in clinical practice to detect atrial fibrosis include direct detection of fibrosis using late gadolinium enhancement (LGE) displayed by cardiac magnetic resonance (CMR), as well as indirect detection of fibrosis using low voltage areas (LVA) on electroanatomic mapping (EAM) and LA strain measured by speckle tracking echocardiography (STE) (9, 223).

### 5.1 LGE magnetic resonance imaging (LGE MRI)

The LGE displayed by CMR has long been proven to be useful for quantifying the degree of LA fibrosis in AF patients (224, 225). The visualization principle of atrial fibrosis area is based on altered washout kinetics of gadolinium. Compared with normal myocardial tissue, gadolinium accumulates in the fibrotic area, resulting in high enhancement in this area, while healthy tissue appears as non-enhanced images (226). The degree of atrial fibrosis was quantified using the Utah classification system proposed by Marrouche et al., and divided into four stages based on the proportion of gadolinium enhancement amount to LA wall volume: stage 1 (<10%), stage 2 (≥10% ≤20%), stage 3 (≥ 20% ≤30%), and stage 4 (≥30%) (31). As an evaluation indicator of atrial fibrosis, LGE detected by CMR and LVA on EAM have mutually confirmed (224, 227). Compared with echocardiography and EAM, CMR exhibits unique advantages in evaluating LA fibrosis. It is less likely to be affected by wall tracing errors (strain and strain rate obtained through echocardiography) and tissue contact (EAM), and can more comprehensively capture potential fibrotic lesions (228, 229). However, the clinical popularization of CMR technology faces practical obstacles. Some medical institutions lack MRI equipment or physicians with CMR expertise, greatly limiting the widespread application of this technology (230). Therefore, under limited conditions, applying indirect evaluation methods such as echocardiography and EAM to detect LA fibrosis is a more clinically feasible alternative.

### 5.2 EAM

The presence of LVA on EAM is considered as a surrogate for the detection of LA fibrosis (9, 223). LA voltage maps are created through thousands of voltage points mapped onto the atrial endocardium's geometric model.LA bipolar voltage amplitude measured by voltage maps is taken to define LVA, characterizing LA fibrosis (231). LVA is usually defined as a bipolar voltage amplitude of less than 0.5 mV (232, 233). However, the voltage threshold of LVA has not been histologically validated (231). Although there is a lack of histological evidence linking LVA to LA fibrosis, previous studies have revealed a high consistency between LVA displayed on EAM and fibrosis areas quantified by LGE MRI (224, 234, 235). It is known that the voltage mapping collected by EAM has some limitations, mainly because the voltage signals change with changes in cycle length and direction of wavefront caused by electrode position, size, and spacing, as well as tissue contact (229, 236).

Substrate mapping based solely on LA LVA measured by EAM cannot fully and accurately quantify LA fibrosis. Regarding this, some scholars have proposed the concept of LA spatial entropy (LASE) measured by EAM, attempting to further characterize LA fibrosis. In the field of cardiac research, entropy can be used to analyze the homogeneity of cardiac tissue and predict related cardiac events (237). There are currently research reports on Shannon entropy, a signal amplitude distribution index, which can be used to measure signal complexity of atrial electrograms, assist AF rotor mapping, assess the nature of AF rotors (238–240). The concept of entropy can also be applied to LA electrical activities. If the amplitude range of atrial voltage is uniform, then entropy will be high. On the contrary, if there is fibrosis present, the distribution of electrical activities will be uneven, leading to skewed probability distribution and a decrease in entropy (237). Gigli et al. demonstrated on this basis that LASE can clearly distinguish between paroxysmal and persistent AF, as well as normal and abnormal LA fibrotic substrates, and is independent of heart rhythm during map collection (241). LASE is a highly sensitive and specific measurement tool that can serve as an auxiliary tool for predicting fibrosis substrates based on EAM.

### 5.3 Two-dimensional (2D) STE

LA strain has been widely recognized as a key indicator for evaluating LA myocardiac deformation (242), and its measurement can be achieved through feasible and reproducible 2D STE (243). In recent years, further research has found that LA strain can also serve as an emerging tool for evaluating LA function (244, 245). Scholars have confirmed that in AF patients, the degree of LA wall fibrosis displayed by LGE MRI was negatively correlated with LA longitudinal strain and strain rate measured by 2D STE (246, 247). In addition, studies have pointed out that cine CMR can also be used for myocardiac feature tracking to quantify LA longitudinal strain and strain rate (248, 249). It should be noted that LA strain and strain rate as alternative indicators for evaluating LA fibrosis also has some drawbacks, such as vendor dependence in LA strain measurement, lack of recognized LA strain reference values, technical bottlenecks in STE image acquisition, etc. (226, 250–252).

## 6 Strategies for treating AF

Targeted intervention in fibrosis and inflammation is a promising treatment strategy for AF, mainly including catheter ablation, RAAS inhibition, anti-inflammatory therapy, lifestyle changes and risk factor management.

### 6.1 Catheter ablation

In recent years, catheter ablation has been increasingly used in the clinical treatment of AF and is the most effective means of rhythm control for AF (253). Pulmonary vein isolation (PVI) is the foundation of catheter ablation. Although a simple PVI strategy can treat most patients with paroxysmal AF, patients with persistent AF who receive catheter ablation therapy face problems such as recurrent arrhythmias after ablation, low long-term success rates, and the need for repeat ablation (254–256).

Atrial fibrosis is an important predictor of poor response to PVI ablation for AF (31, 257). There are significant differences in the localization and degree of LA fibrosis among AF patients, which can serve as individual fingerprints reflecting potential arrhythmogenic substrates. Therefore, accurate localization and quantification of atrial fibrosis may provide strong support for personalized ablation strategies in AF patients (258). We have previously described in detail the techniques used clinically to detect atrial fibrosis, including LGE MRI, EAM, and STE. These tools can be used to supplement PVI strategies to improve the effectiveness of catheter ablation. Some research reported that targeted therapy for atrial fibrosis detected by LGE MRI is a novel custom-tailored ablation strategy for treating recurrent arrhythmia after AF ablation (258, 259). Many researchers have demonstrated that fibrotic substrate modification based on LA LVA detected by EAM is a new assistant technology for PVI ablation. Compared with AF patients who only received PVI ablation, patients who received further LVA guided substrate modification had a significantly lower recurrence rate of AF (260, 261). This indicates that this personalized arrhythmogenic substrate modification can effectively improve the prognosis of PVI ablation in AF patients. Kottkamp et al. applied a patient-tailored modification strategy targeting fibrotic substrates to AF patients undergoing catheter ablation: box isolation of fibrotic areas. This strategy provides a new treatment option for AF patients undergoing simple PVI ablation by performing circumferential isolation on EAM characterized fibrotic substrates (LVA: <0.5 mV) (262). Clarifying the individual distribution and quantity of LA fibrotic substrates may provide personalized ideas for the prevention, diagnosis, and treatment of AF patients. For example, the burden of LA fibrosis in AF patients can be included in the AF risk stratification and staging system. The substrate modification targeting LA fibrosis can be applied as a supplementary strategy for PVI ablation. However, these ideas need to be confirmed and validated for their effectiveness in prospective, multi-center, randomized clinical studies.

Interestingly, the clinical outcomes of AF ablation show significant sex differences. Compared with male AF patients, women who undergo catheter ablation have a higher risk of arrhythmia recurrence, lower rates of arrhythmia free survival, and increased risks of postoperative complications and rehospitalization (263–265). These observations suggest that the AF mechanism may vary by gender. LGE MRI showed that women had a greater burden of atrial fibrosis compared to men (266). A histological analysis involving fibrosis markers also showed that women had a higher degree of atrial fibrosis than men (267). In addition, LA LVA measured by EAM was more likely to occur in females than males, which was a powerful predictor of AF recurrence after ablation (268). Based on this, Wong et al. demonstrated significant sex differences in atrial electrophysiology in AF patients using high-density EAM, characterized by female AF patients having more advanced atrial substrates, including lower voltage, slower conduction velocity, and a higher proportion of complex fractionated potentials (269). The above research provides important reference for conducting gender specific risk stratification and developing personalized ablation strategies in clinical practice, helping optimize the diagnosis and treatment protocols for AF patients of different genders.

### 6.2 RAAS inhibition

As discussed earlier, RAAS activation can promote the formation of atrial fibrosis (270–272). Multiple studies have shown that ACEIs, angiotensin II receptor blockers (ARBs), and mineralocorticoid receptor antagonists (MRAs) reduce the progression of atrial fibrosis by inhibiting RAAS activation, thereby treating AF (37, 44, 273–277). In addition, RAAS activation has also been shown to be closely related to inflammation (278–280). Studies have shown that ACEI/ARB can effectively reduce levels of pro-inflammatory cytokines (281, 282), which may be a mechanism for treating AF (283). Sacubitril/valsartan (Sc/Pal) is currently a relatively new drug for treating HF (284). Sc/Pal can simultaneously antagonize angiotensin receptors and neprilysins, exerting anti-inflammatory, anti-fibrotic, and anti-hypertrophic effects by blocking AT1R and inhibiting natriuretic peptide degradation (285–288). It was showed that Sc/Pal can improve left atrial and left atrial appendage function in patients with AF and pressure-overloaded mice, which may be a new approach for treating atrial remodeling and AF (289).

### 6.3 Anti-inflammatory therapy

Multiple clinical studies have confirmed that some anti-inflammatory drugs, such as steroids (290–294), colchicine (295–299), and statins (300–305), can effectively prevent the recurrence of AF after ablation or cardioversion, as well as new-onset AF after cardiac surgery. In addition, drugs targeting inflammatory cytokines are gradually being applied in cardiovascular and cerebrovascular diseases (306–309). However, potential risks in clinical application require vigilance. A case report documented recurrent AF episodes in a multiple sclerosis patient following high-dose methylprednisolone (a glucocorticoid) treatment (310). While colchicine reduces AF recurrence rates, it increases gastrointestinal adverse effects (311). Although these anti-inflammatory drugs show promise in cardiovascular disease treatment, their precise efficacy in AF management requires further research validation. Clinical practice should incorporate individualized risk-benefit assessments based on patient characteristics. Future studies need to further elucidate drug mechanisms and optimize treatment protocols to achieve safe and effective personalized therapy.

### 6.4 Lifestyle changes and risk factor management

Inflammation related risk factors of AF include obesity, lack of exercise, hypertension, diabetes, sleep apnea, smoking/drinking habits (138, 312). Multiple studies have confirmed that managing the above risk factors can effectively prevent the occurrence of AF, help AF patients reduce the burden of AF symptoms, maintain SR, and reduce AF recurrence (313–317).

## 7 Conclusion

The pathophysiology of AF is very complex, and atrial fibrosis and inflammation play key roles in it. Atrial fibrosis and inflammation are simultaneous and mutually reinforcing processes in the occurrence and development of AF, and they synergistically promote atrial remodeling, leading to the occurrence and persistence of AF. A better understanding of the role, characteristics, and mechanisms of atrial fibrosis and inflammation during AF may help identify new clinical biomarkers and develop new, personalized, and more effective treatments for AF.
